# Non-Invasive Composition Identification in Organic Solar Cells via Deep Learning

**DOI:** 10.3390/nano15141112

**Published:** 2025-07-17

**Authors:** Yi-Hsun Chang, You-Lun Zhang, Cheng-Hao Cheng, Shu-Han Wu, Cheng-Han Li, Su-Yu Liao, Zi-Chun Tseng, Ming-Yi Lin, Chun-Ying Huang

**Affiliations:** 1 Department of Applied Materials and Optoelectronic Engineering, National Chi Nan University, Nantou 54561, Taiwan; yihsun0716@gmail.com (Y.-H.C.); a0956593898@gmail.com (Y.-L.Z.); suyu@ncnu.edu.tw (S.-Y.L.);; 2 Department of Electrical Engineering, National United University, Miaoli 360302, Taiwan; bob30610@gmail.com (C.-H.C.); qwer2623260@gmail.com (S.-H.W.); hank977@gmail.com (C.-H.L.)

**Keywords:** organic photovoltaic (OPV), absorption spectra, deep learning, multilayer perceptron (MLP), non-invasive classification

## Abstract

Accurate identification of active-layer compositions in organic photovoltaic (OPV) devices often relies on invasive techniques such as electrical measurements or material extraction, which risk damaging the device. In this study, we propose a non-invasive classification approach based on simulated full-device absorption spectra. To account for fabrication-related variability, the active-layer thickness varied by over ±15% around the optimal value, creating a realistic and diverse training dataset. A multilayer perceptron (MLP) neural network was applied with various activation functions, optimization algorithms, and data split ratios. The optimized model achieved classification accuracies exceeding 99% on both training and testing sets, with minimal sensitivity to random initialization or data partitioning. These results demonstrate the potential of applying deep learning to spectral data for reliable, non-destructive OPV composition classification, paving the way for integration into automated manufacturing diagnostics and quality control workflows.

## 1. Introduction

Organic photovoltaic (OPV) devices represent a compelling avenue within third-generation solar cell technologies due to their distinct advantages, including solution-processable fabrication, mechanical flexibility, and lightweight form factor [1,2]. These characteristics make OPVs particularly well-suited for scalable, low-energy manufacturing and integration into next-generation flexible or wearable electronic systems [3]. Structurally, OPVs are broadly categorized into conventional and inverted configurations, distinguished by whether the hole transport layer (HTL) or the electron transport layer (ETL) is deposited directly onto the bottom electrode, typically composed of indium tin oxide (ITO) [4]. This architectural choice significantly influences charge extraction dynamics, interfacial energy alignment, and the overall operational stability of the device [4]. Various strategies have been employed to improve the power conversion efficiency (PCE) of OPVs, including optimization of charge transport layers, tuning of electrode work functions, and engineering of interfacial morphology [5]. Among these, the most direct and impactful remains the development of new donor–acceptor material systems [6]. In particular, combining conjugated polymers with non-fullerene acceptors (NFAs) has significantly enhanced device performance by broadening the optical absorption range and enabling more favorable energetic alignment [7,8]. However, as the diversity of donor–acceptor combinations continues to expand, identifying the exact composition of a fabricated device has become increasingly challenging [9]. This challenge is particularly pronounced in real-world scenarios, where device encapsulation, structural similarities among materials, and high-throughput production requirements limit the applicability of destructive or time-consuming characterization techniques [10,11].

In practice, even experienced researchers often rely on current–voltage measurements to infer material composition based on differences in open-circuit voltage or short-circuit current density [12]. Yet such methods are not truly non-invasive: many active-layer materials undergo irreversible changes when exposed to ambient conditions or handled outside a glovebox, and perovskite- or polymer-based devices frequently exhibit hysteresis or degradation upon repeated testing [13]. These limitations suggest that conventional electrical characterization may inadvertently alter the very properties it seeks to probe. The need for non-destructive identification methods is further amplified in various real-world scenarios. In large-scale manufacturing, quality control personnel may need to verify the active-layer composition of encapsulated OPV modules without damaging the devices, particularly in cases of suspected material mix-ups or process deviations [14,15]. In academic laboratories, improperly labeled or long-stored “black-box” devices often require retrospective identification when original fabrication records are missing or incomplete. Similarly, third-party testing laboratories or clients may request composition verification of commercial OPV samples as part of product authentication or certification workflows. In all such cases, a fast, reliable, and non-invasive method for determining active-layer composition would be highly valuable for research, production, and quality assurance contexts [16].

Recent advances in deep learning have transformed the landscape of spectral analysis across a range of disciplines, including chemical sensing, biomedical diagnostics, and remote sensing [17]. Unlike traditional machine learning techniques that rely heavily on handcrafted feature extraction and domain-specific preprocessing, neural networks can automatically learn hierarchical representations directly from raw spectral data [17]. This end-to-end learning capability enables deep learning models to capture subtle, nonlinear patterns that may be overlooked by conventional methods, facilitating rapid, high-throughput analysis with minimal human intervention [18]. For example, multilayer perceptron (MLP) architectures have proven effective in modeling complex nonlinear relationships in spectral data [19]. Owing to their simplicity, fast training, and compatibility with one-dimensional inputs, MLPs are particularly well-suited for absorption spectrum classification tasks, especially when informative features are distributed across the entire wavelength range [20]. Despite this progress, the application of deep learning to classify OPV devices based solely on their absorption spectra remains largely unexplored.

In this study, we propose a data-driven, non-invasive framework that establishes a robust mapping between the full absorption spectrum (350–750 nm) and the specific donor–acceptor pair used in the OPV active layer, even under realistic fabrication variations in layer thickness. Using an optical modeling approach, we systematically generated spectra for six representative donor–acceptor systems, namely P3HT:PCBM, PTB7-Th:PCBM, PBDB-T:IT4F, PBDB-T:ITIC, PM6:Y6, and PM6:IT4F, incorporating process-induced deviations to ensure that the training data reflect practical manufacturing conditions. A multilayer perceptron (MLP) model was then developed, and its performance was rigorously evaluated across various activation functions, hidden neuron counts, random seeds, and train–test splits. The model successfully learns to distinguish the unique spectral fingerprint of each composition despite spectral overlap and thickness-induced noise, demonstrating the feasibility of applying deep learning for rapid, non-destructive composition identification and paving the way for reliable integration into automated quality control workflows for organic solar cell production.

## 2. Experimental

### 2.1. Device Fabrication

**Figure 1:**
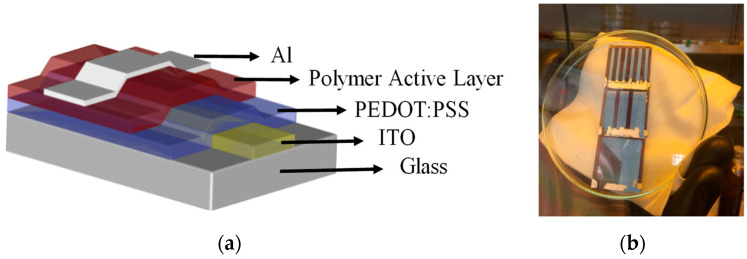
(**a**) Schematic structure of the fabricated OPV device, consisting of a multilayer stack including ITO, PEDOT:PSS, P3HT:PCBM, LiF, and Al. (**b**) Photograph of completed OPV devices inside the nitrogen-filled glovebox.

Standard OPV devices were fabricated on pre-patterned ITO-coated glass substrates with dimensions of 2.0 cm × 2.0 cm × 1.1 mm and a sheet resistance of 15 Ω/sq. The substrates were sequentially cleaned by sonication in detergent solution, deionized water, acetone, and isopropanol, followed by ultraviolet-ozone treatment to enhance surface wettability and promote uniform film deposition. A filtered solution of PEDOT:PSS (Baytron P Al4083) was spin-coated at 3000 rpm for 60 s to form a ~35 nm-thick HTL, followed by thermal annealing at 130 °C for 10 min in a nitrogen-filled glovebox to remove residual solvent and moisture. All subsequent steps were performed under an inert nitrogen atmosphere. The photoactive layer was prepared by dissolving P3HT and PCBM in a 1:1 weight ratio to make a 3 wt% solution in ortho-dichlorobenzene. The solution was stirred overnight at 80 °C and filtered through a PTFE membrane prior to deposition. The active layer was spin-coated at 600 rpm for 40 s and subsequently covered with a Petri dish for 20 min to induce solvent annealing. A second thermal annealing step at 130 °C for 10 min was applied to promote phase separation and improve film morphology. Next, a 1 nm LiF ETL and a 150 nm Al top electrode were deposited sequentially by thermal evaporation at a base pressure of ~10–7 mbar and a deposition rate of ~1.5 Å/s through a shadow mask, without breaking vacuum. The device’s active area was defined by the overlap between the ITO bottom electrode and the Al top electrode, as illustrated in Figure 1a. A representative image of the completed OPV cells inside the glovebox is shown in Figure 1b, confirming precise electrode alignment and clean processing conditions. These devices were subsequently characterized by optical transmission measurements and used to validate the deep learning classification model.

### 2.2. Optical Simulation

To generate the spectral dataset for deep learning classification, we employed Fluxim Setfos^®^ 5.5, a commercial optical simulation tool specialized for thin-film optoelectronic devices. The software uses the transfer matrix method (TMM) to solve Maxwell’s equations for multilayer stacks, enabling accurate modeling of wavelength-dependent reflection, transmission, and absorption under coherent illumination [21]. To ensure consistent modeling conditions, the layer stack for all devices was based on a standard conventional architecture (glass/ITO/PEDOT:PSS/active layer/LiF/Al), with fixed thicknesses for ITO (150 nm), PEDOT:PSS (35 nm), LiF (1 nm), and Al (150 nm). Six representative donor–acceptor material pairs were investigated as the active layer, including P3HT:PCBM and PTB7-Th:PCBM as typical fullerene-based systems, and PBDB-T:IT4F, PBDB-T:ITIC, PM6:Y6, and PM6:IT4F as modern non-fullerene acceptor configurations. This diverse selection provides distinct yet partially overlapping spectral features, enabling the machine learning model to learn robust classification boundaries for reliable composition identification. All optical simulations were performed using the transfer matrix method with complex refractive index (n, k) data imported from experimental sources, ensuring realistic optical behavior for each material layer [22]. Simulations were carried out under normal incidence and ambient conditions, assuming coherent optical propagation throughout all layers. For each donor–acceptor material combination, we first determined the optimal active-layer thickness based on a peak photocurrent under AM1.5G illumination, as shown in Figure S1 [23,24]. Around this nominal thickness, controlled variations were introduced to reflect realistic fabrication tolerances. Specifically, the active-layer thickness varied from −20 nm to +20 nm in 1 nm increments relative to the nominal value. These spectra effectively capture the subtle influence of thickness-induced interference effects on optical performance while maintaining a consistent device architecture and material composition. This systematic simulation procedure yielded a high-quality, label-rich dataset suitable for deep learning models. By embedding fabrication-relevant variations into the synthetic spectra, the resulting dataset enables the model to learn robust features that generalize beyond idealized conditions, supporting reliable post-fabrication classification of OPV device types.

### 2.3. Data Processing Using Deep Learning

**Figure 2:**
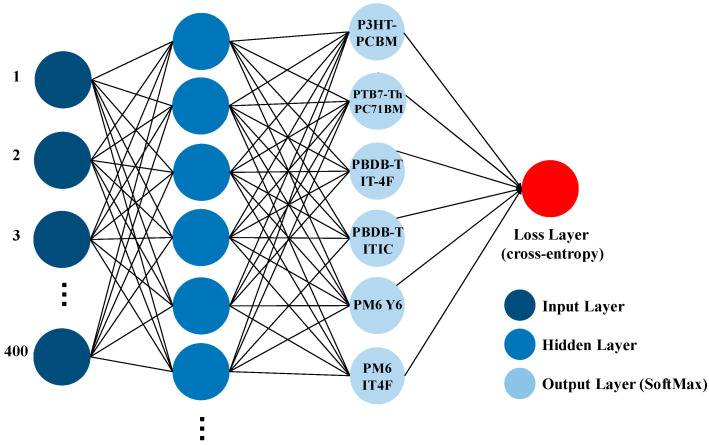
Schematic diagram of the multilayer perceptron (MLP) architecture used for absorption spectrum-based classification of OPV device compositions. The input layer consists of 400 spectral data points (350–750 nm), the hidden layer has tunable neurons and activation functions, and the output layer employs a SoftMax classifier to identify one of six donor–acceptor pairs.

To classify different types of OPV devices based on their active-layer composition, we implemented an MLP neural network using STATISTICA 14 software. The input data consisted of simulated full-device absorption spectra generated under realistic fabrication conditions, with active-layer thickness variations incorporated to reflect process-induced fluctuations. Each spectrum contained 400 data points, uniformly sampled from 350 nm to 750 nm at 1 nm intervals. The MLP architecture was constructed as a fully connected feedforward network, enabling the model to learn nonlinear mappings between optical features and categorical OPV types. Model training was initially performed using the standard gradient descent algorithm, during which key hyperparameters, such as the number of hidden neurons (ranging from 2 to 16), were systematically tuned. Various activation functions were tested, including Identity, Logistic, Tanh, Exponential, and Sine. In addition to gradient descent, we employed the Broyden–Fletcher–Goldfarb–Shanno (BFGS) optimization method to improve training efficiency and robustness. Both optimization approaches were evaluated across multiple train–test split ratios (70:30, 80:20, and 90:10), and repeated using different random seed values to assess model stability and generalization. A schematic representation of the MLP classification framework is shown in Figure 2.

## 3. Results and Discussion

### 3.1. Absorption Spectra Analysis

**Figure 3:**
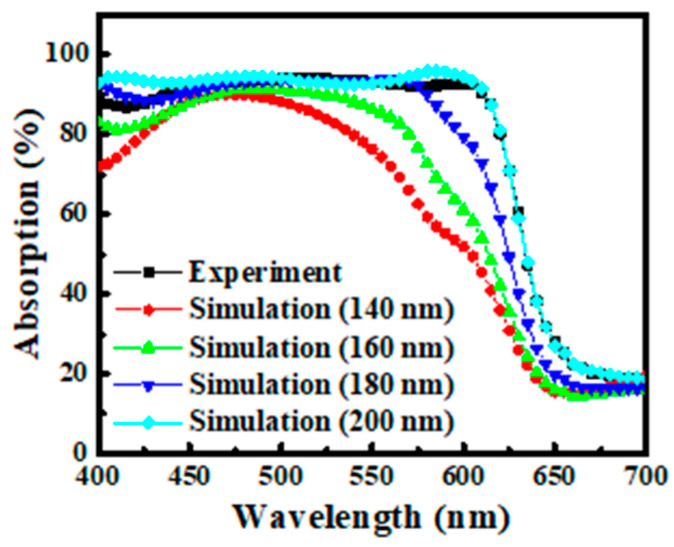
Comparison between the experimentally measured absorption spectrum of the fabricated P3HT:PCBM OPV device and simulated spectra under varying active-layer thicknesses (140–200 nm).

Figure 3 shows the experimentally measured absorption spectrum of the fabricated OPV device alongside simulated spectra at different active-layer thicknesses, all based on the same device architecture and P3HT:PCBM blend. Optical transmission spectra were recorded using a UV−2501PC UV–visible spectrophotometer (Princeton Instruments, Trenton, NJ, USA) under normal incidence in the wavelength range of 350–750 nm. A cleaned bare glass substrate was used as the reference for baseline correction.

The spectral profiles across simulations exhibit consistent features in the 450–650 nm region, aligning with the known optical characteristics of the donor–acceptor system. By systematically varying the active-layer thickness from 140 nm to 200 nm, we found that the simulated spectrum at 200 nm most closely matched the experimental data. This strong agreement underscores the accuracy of the optical simulation and confirms that layer thickness is a dominant factor influencing absorption spectra. These findings demonstrate that, when thickness is carefully calibrated, optical simulations can reliably reproduce real device behavior and provide a robust foundation for synthetic data generation in deep learning applications.

**Figure 4:**
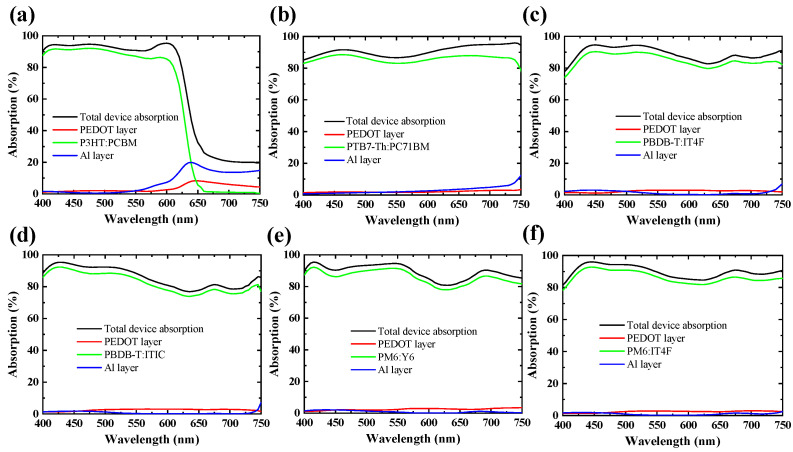
Simulated absorption spectra of organic photovoltaic (OPV) devices incorporating six representative donor–acceptor combinations: (**a**) P3HT:PCBM, (**b**) PTB7-Th:PCBM, (**c**) PBDB-T:IT4F, (**d**) PBDB-T:ITIC, (**e**) PM6:Y6, and (**f**) PM6:IT4F. All devices share a conventional OPV multilayer structure with fixed thicknesses for ITO (150 nm), PEDOT:PSS (35 nm), LiF (1 nm), and Al (150 nm).

Figure 4 presents the simulated absorption spectra of OPV devices incorporating six representative donor–acceptor combinations: P3HT:PCBM, PTB7-Th:PCBM, PBDB-T:IT4F, PBDB-T:ITIC, PM6:Y6, and PM6:IT4F. These systems span both classic fullerene-based architectures and modern NFA configurations, covering a broad spectral range and diverse absorption characteristics. All simulations were performed using a conventional OPV stack with fixed layer thicknesses for ITO (150 nm), PEDOT:PSS (35 nm), LiF (1 nm), and Al (150 nm). The active-layer thickness was systematically varied from 80 nm to 300 nm to determine the condition yielding the highest simulated photocurrent for each material combination. The absorption spectra shown in Figure 4 correspond to these optimized thicknesses. Although differences in absolute absorption intensity are observed, the spectral shapes reveal material-specific features. For instance, P3HT:PCBM displays a distinctly shorter absorption cutoff near 650 nm [25], while systems such as PM6:Y6 and PBDB-T:IT4F exhibit broader absorption extending beyond 800 nm [26]. These distinctions reflect the intrinsic optical properties of the donor–acceptor pairs and support the hypothesis that full-device absorption spectra retain unique compositional signatures. The unique absorption features arise from the distinct donor and acceptor bandgaps, optical transition energies, and extinction coefficients, which collectively determine the wavelength-dependent absorption strength [23,24]. However, when these optimized spectra are overlaid, significant spectral overlap emerges among most non-fullerene systems. This is largely due to the fact that only one component differs in many material pairs, resulting in similar spectral profiles. Even experienced researchers may find it challenging to distinguish these systems based solely on visual inspection. In contrast, contributions from other device layers (e.g., PEDOT:PSS or Al/LiF) appear to have a relatively minor effect within this wavelength range, further emphasizing the dominant role of the active layer in shaping the spectral response. So, the overall device spectra closely resemble the inherent active-layer absorption profiles, and these embedded material-specific optical fingerprints can be effectively leveraged by the machine learning model for accurate and robust composition classification.

To incorporate fabrication-relevant variability into the training dataset, we further simulated the absorption spectra of all six OPV material systems over a range of active-layer thicknesses centered around their respective optimal values. For each system, the thickness varied from −20 nm to +20 nm in 1 nm increments. Although 1 nm differences may not lead to large spectral changes individually, they can influence optical interference effects in multilayer stacks and serve as a valuable source of variation for data augmentation in ML training.

**Figure 5:**
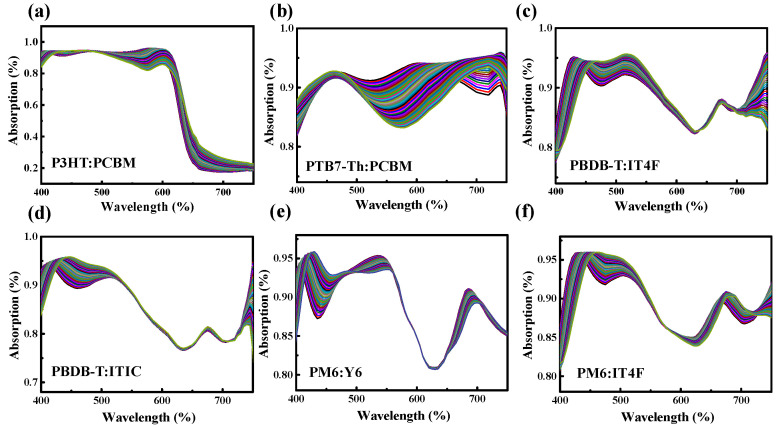
Overlaid simulated absorption spectra for six OPV material systems, each with active-layer thickness varied by ±20 nm in 1 nm increments around its optimized value: (**a**) P3HT:PCBM, (**b**) PTB7-Th:PCBM, (**c**) PBDB-T:IT4F, (**d**) PBDB-T:ITIC, (**e**) PM6:Y6, and (**f**) PM6:IT4F. These spectra form the training dataset for the deep learning classification model.

The overlaid spectra, shown in Figure 5, constitute the full dataset used to train the deep learning classification model. This approach provides two key advantages. First, it reflects the inherent variability of thin-film deposition processes, where precise thickness control is often challenging, particularly in solution-processed organic layers. Even under optimized laboratory conditions, deviations of ±10–20 nm are common due to factors such as solvent evaporation rate, spin-coating dynamics, and ambient temperature or humidity [27]. In large-scale manufacturing, these variations may be even more pronounced. By explicitly incorporating such fluctuations into the training data, the model is trained to generalize across realistic, thickness-induced spectral shifts, rather than overfitting to a single “ideal” condition. Second, as demonstrated in Figure 3, active-layer thickness plays a dominant role in shaping the device’s optical response, significantly affecting both the shape and intensity of the absorption spectrum. Without accounting for this variation, even small thickness differences could introduce spectral distortions that confound material identification. In this context, training with a diversified dataset not only enhances the robustness of the classification model but also ensures that it can tolerate typical deviations encountered in post-fabrication scenarios.

### 3.2. Deep Learning Analysis

**Figure 6:**
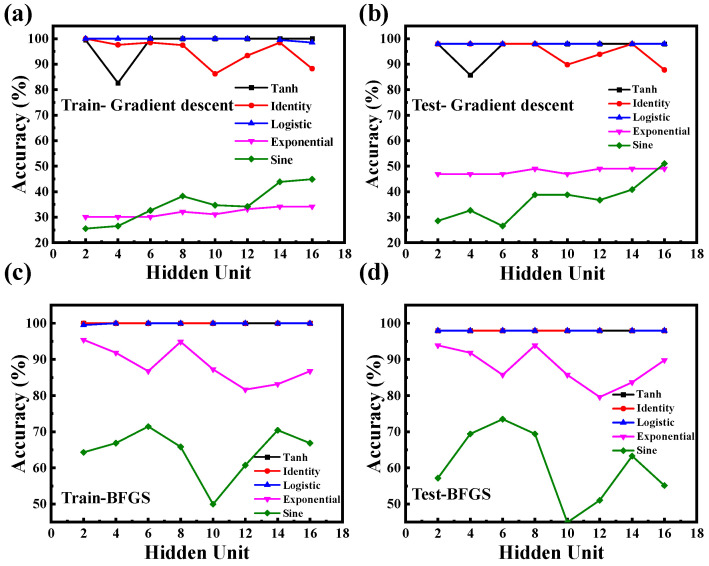
Classification accuracy of the MLP model under various activation functions and hidden neuron counts using two different optimization algorithms. (**a**) Training accuracy using traditional gradient descent. (**b**) Corresponding testing accuracy with traditional gradient descent. (**c**) Training accuracy using the BFGS optimization algorithm. (**d**) Corresponding testing accuracy with the BFGS.

Figure 6 evaluates the classification performance of the MLP model under various activation functions and hidden layer sizes. Figure 6a shows that, when trained using the traditional gradient descent algorithm, logistic and tanh activation functions consistently achieve near-perfect training accuracy across a range of hidden neuron counts [28]. In contrast, identity, sine, and exponential functions yield significantly lower accuracy [28]. This discrepancy can be attributed to differences in the nonlinear mapping capabilities of the activation functions. Both logistic and tanh introduce smooth, bounded nonlinearities and maintain non-zero gradients across a wide input range, thereby facilitating effective backpropagation and representation learning [28]. By comparison, identity lacks nonlinearity, sine may introduce oscillatory behavior that destabilizes training, and exponential activations can lead to exploding gradients [28]. These findings highlight the importance of selecting appropriate activation functions when designing neural architectures for OPV spectral classification.

Figure 6b displays the corresponding prediction accuracies on the testing set. A similar trend is observed, with logistic and tanh again yielding high accuracy. Notably, the close agreement between training and testing results across all configurations indicates that the model does not suffer from overfitting [29]. This suggests that the simulated spectral dataset is sufficiently diverse to support generalizable learning, and that the selected MLP configuration achieves a strong balance between complexity and robustness.

Figure 6c,d present the classification accuracies of the MLP model trained using the BFGS optimization algorithm for the training and testing sets, respectively. Compared to gradient descent, the BFGS demonstrates superior and more consistent performance across all activation functions [30]. Specifically, tanh, identity, and logistic activations all achieve near-perfect classification, exceeding 99% accuracy on both training and testing sets. Even sine and exponential functions show substantial improvement when optimized with the BFGS. This convergence in performance suggests that the BFGS is less sensitive to the choice of activation function and is better suited to optimizing the model’s weight space in this context. The enhanced performance of the BFGS can be attributed to its quasi-Newton optimization approach, which approximates the second-order curvature (Hessian matrix) of the loss surface to guide weight updates [31]. In contrast to basic gradient descent, the BFGS dynamically adjusts both the search direction and step size, enabling faster and more stable convergence. This is particularly advantageous in spectral classification tasks, where the loss landscape is often highly nonlinear and sensitive to initialization. Among all tested configurations, the logistic activation function consistently achieved perfect classification accuracy across training and testing sets, regardless of the optimizer or hidden-layer size. This suggests that logistic is particularly well-suited for the spectral classification task due to its stable gradients and bounded nonlinear characteristics.

### 3.3. Confusion Matrix Analysis

**Figure 7:**
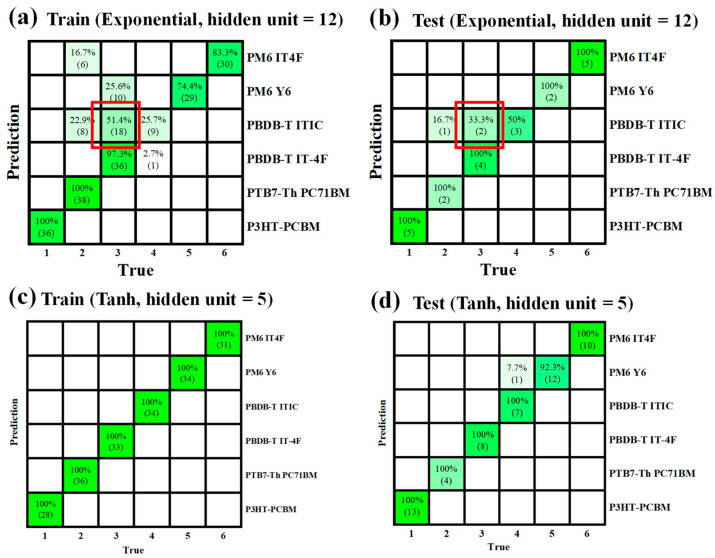
Confusion matrices illustrating representative classification results under different MLP configurations. (**a**) Training and (**b**) testing set confusion matrix using the exponential activation function with 12 hidden neurons. (**c**) Training and (**d**) testing set confusion matrix using the tanh activation function with 5 hidden neurons.

Figure 7 illustrates confusion matrices under two representative modeling conditions. Specifically, Figure 7a presents the confusion matrix for the training set using the exponential activation function with 12 hidden neurons, while Figure 7b shows the corresponding result for the testing set. In both cases, diagonal elements represent correctly classified instances, whereas off-diagonal entries indicate misclassifications. Several off-diagonal errors are observed, most notably between PBDB-T:ITIC and PBDB-T:IT4F. These misclassifications highlight the inherent difficulty in distinguishing material pairs with highly similar optical absorption spectra. This observation aligns with the spectral trends shown earlier in Figure 4c,d for PBDB-T:IT4F and PBDB-T:ITIC, where the absorption profiles are nearly indistinguishable in both shape and spectral range. Such spectral resemblance introduces intrinsic ambiguity in classification, particularly when suboptimal model configurations are used, such as the exponential activation function in this case. However, when a more suitable architecture is employed (e.g., tanh activation with 5 hidden neurons), the resulting confusion matrix shows a marked reduction in misclassification, with most predictions concentrated along the main diagonal. This outcome suggests that the careful selection of activation function and model architecture is crucial for maximizing classification accuracy, especially when differentiating between spectrally similar systems.

### 3.4. Robustness Evaluation

**Figure 8:**
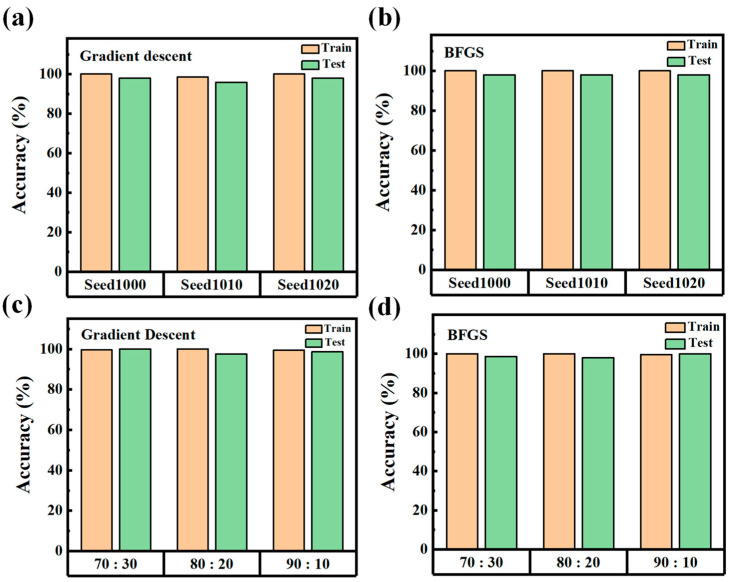
Robustness evaluation of the MLP classification model using the optimized configuration (tanh activation, 5 hidden neurons). (**a**) Prediction accuracies on the training and testing sets using gradient descent across different random seed values. (**b**) Corresponding results using the BFGS optimization algorithm. (**c**) Prediction accuracy using gradient descent under different training/testing split ratios (70:30, 80:20, and 90:10). (**d**) Corresponding performance using the BFGS under the same data splits.

Figure 8 evaluates the robustness of the MLP classification model under varying random seed initializations and different training/testing split ratios [32,33]. All results were obtained using the optimized network configuration with the tanh activation function and 5 hidden neurons. Figure 8a shows the prediction accuracies on the training and testing sets using the gradient descent algorithm across different random seeds, while Figure 8b presents the corresponding results using the BFGS optimization method. In both cases, the model consistently achieves high accuracy with minimal variation, indicating strong resilience to the stochastic nature of weight initialization and data shuffling. This confirms the model’s ability to converge reliably to an effective solution regardless of initial parameter conditions. Figure 8a,d explore the effect of varying training-to-testing data split ratios, specifically 70:30, 80:20, and 90:10, on model performance using gradient descent and the BFGS, respectively. Across all tested splits, classification accuracy remains near 100% for both training and testing sets. This result demonstrates strong generalization capability, even when the training set is relatively small. The model’s consistent performance across different data proportions further validates the robustness of the selected MLP architecture. Overall, these findings indicate that the deep learning framework offers a highly stable and reproducible solution for classifying OPV compositions from absorption spectra. The model is not only insensitive to typical sources of variability but also maintains near-perfect performance across multiple optimization strategies. Such robustness is critical for real-world deployment in manufacturing or diagnostic settings, where input fluctuations and limited data availability are common. Considering both accuracy and deployment efficiency, the MLP model trained using gradient descent is recommended. This configuration achieves near-perfect classification while minimizing computational load, making it ideal for edge deployment scenarios.

Although the present study relies on simulated spectral data for model training, this choice was deliberate and strategic. Simulation allows us to precisely control material combinations and fabrication parameters, enabling the creation of a diverse and balanced dataset that would be difficult to achieve through purely experimental means. In particular, it enables systematic modeling of thickness variations and idealized optical responses, free from measurement noise or fabrication artifacts. This provides a clean benchmark for validating the deep learning architecture. As the next step, this framework can be adapted and retrained on real-world experimental data, thereby bridging the gap between theoretical model development and practical deployment. To further enhance its utility in practical OPV design, future extensions may also incorporate photovoltaic performance metrics, such as conversion efficiency, into the machine learning pipeline, enabling not only material classification but also performance-aware screening and optimization.

## 4. Conclusions

This study presents a deep learning framework for classifying OPV device compositions based on simulated absorption spectra, incorporating ±15% variation in active-layer thickness to reflect practical fabrication tolerances. Using an MLP architecture, we systematically evaluated multiple activation functions and optimization algorithms, with the optimal configuration achieving classification accuracies exceeding 99% on both training and testing sets. Confusion matrix analysis confirmed the model’s ability to resolve spectrally similar material systems when appropriately configured. While simulated data were used to provide a controlled and label-rich environment for model development, the approach establishes a transferable foundation for future deployment with experimental datasets. The ability of the model to generalize across spectral variations suggests strong potential for integration into real-time, non-invasive quality control workflows. Overall, this work demonstrates the feasibility of using deep learning for optical classification in OPV systems and paves the way toward robust, data-driven diagnostics in next-generation photovoltaic technologies. This robust spectrum-to-composition mapping framework provides a practical foundation for integrating non-invasive composition verification into real-time quality control workflows in OPV manufacturing. Future work will focus on expanding the dataset to cover a wider range of donor–acceptor systems and validating the model performance using experimentally measured spectra for large-scale manufacturing applications.

## Data Availability

Data are contained within the article and Supplementary Materials.

## Supplementary Materials

The following supporting information can be downloaded at: https://www.mdpi.com/article/10.3390/nano15141112/s1, Figure S1: Simulated photocurrent density as a function of active-layer thickness for six representative donor–acceptor systems. For each material, the maximum photocurrent condition is identified and used to generate the optimized absorption spectra presented in Figure 3. The simulations confirm that optimal active-layer thicknesses vary by material, typically within the range of 200–300 nm.
