# Supplementary material for: Non-Invasive Composition Identification in Organic Solar Cells via Deep Learning
Source: Nanomaterials (Basel). 2025 Jul 17;15(14):1112. doi: 10.3390/nano15141112 (PMC12299612; doi:10.3390/nano15141112)
Supplement: Supplementary file 1 [file nanomaterials-15-01112-s001.zip › nanomaterials-3700176-supplementary.pdf]

Supplementary material

Non-Invasive Composition Identification in Organic Solar Cells via Deep Learning

Yi-Hsun Chang[1], You-Lun Zhang[1], Cheng-Hao Cheng[2], Shu-Han Wu[2], Cheng-Han Li[2], Su-Yu Liao[1], Zi-Chun Tseng[1], Ming-Yi Lin[2,*], and Chun-Ying Huang[1,*]

[1] Department of Applied Materials and Optoelectronic Engineering, National Chi Nan University, Nantou 54561, Taiwan
[2] Department of Electrical Engineering, National United University, Miaoli 360302, Taiwan

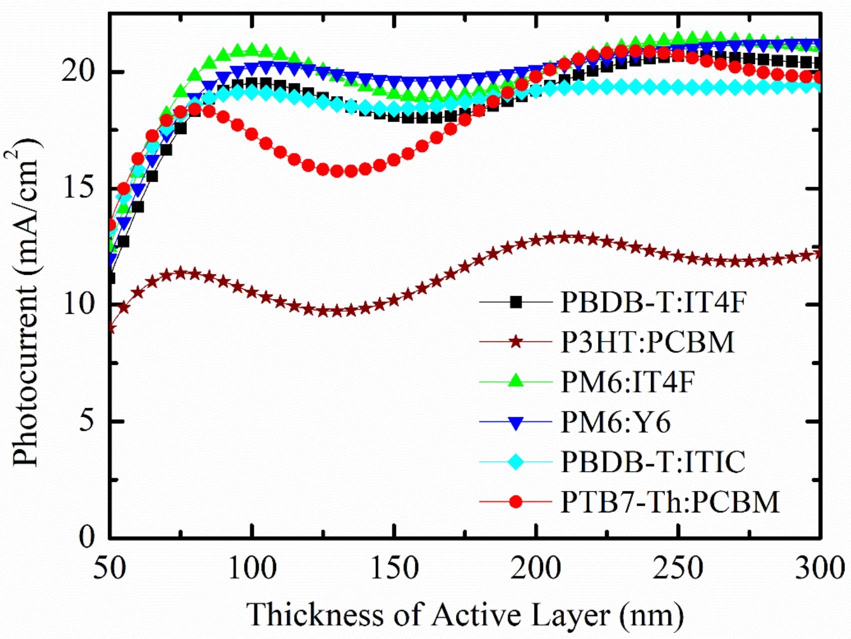

Figure S1. Simulated photocurrent density as a function of active-layer thickness for six representative donor–acceptor systems. For each material, the maximum photocurrent condition is identified and used to generate the optimized absorption spectra presented in Figure 3. The simulations confirm that optimal active-layer thicknesses vary by material, typically within the range of 200–300 nm.
